# Toward precision psychiatry: theoretical implications of bimodal response patterns to vasopressin V1b receptor inhibition in depression

**DOI:** 10.3389/fpsyt.2025.1645225

**Published:** 2025-10-16

**Authors:** Christine zu Eulenburg, Daniel Gehrlach, Marius Myhsok, Caren Strote, Lars Arvastson, Bertram Mueller-Myhsok, Guy Griebel, Hans Eriksson

**Affiliations:** ^1^ HMNC Holding GmbH, Munich, Germany; ^2^ Department for Medical Biometry and Epidemiology, University Medical Center Hamburg-Eppendorf, Hamburg, Germany; ^3^ Ludwig-Maximilian Universität (LMU) University Hospital, LMU Munich, Munich, Germany; ^4^ Max-Planck-Institute of Psychiatry, Munich, Germany; ^5^ Sanofi, Paris, France

**Keywords:** major depressive disorder, MDD, HPA-axis, vasopressin V1b receptor antagonists, nelivaptan, finite mixtures models, FMM, Hamilton Depression Rating Scale

## Abstract

**Introduction:**

Despite extensive research and numerous available treatments, major depressive disorder (MDD) remains a significant global health issue with limited efficacy from current monoaminergic antidepressants. Dysfunction in the hypothalamic–pituitary–adrenal (HPA) axis has been implicated in a subgroup of depressed patients, leading to interest in developing targeted treatments such as vasopressin V1b receptor antagonists. Nelivaptan, a potent V1b antagonist, demonstrated statistically significant antidepressant efficacy in one of two previous Phase 2 trials but was not pursued further.

**Methods:**

We reanalyzed the trial data (NCT00358631) using a finite mixture of linear regression model (FMM) to investigate whether antidepressant responses to nelivaptan exhibit a bimodal distribution, suggesting distinct responder subgroups. We analyzed the 17-item Hamilton Rating Scale for Depression (HAMD) scores from baseline to day 56 for patients treated with 250 mg BID nelivaptan (n = 62) versus placebo (n = 63).

**Results:**

Our analyses revealed a bimodal response distribution exclusively in the nelivaptan-treated group, characterized by two distinct subpopulations: a high-responder subgroup (mean change: −17.14) and a low-responder subgroup (mean change: −3.85). In contrast, the placebo group displayed a unimodal distribution (mean change: −7.06).

**Discussion:**

These findings support the hypothesis that nelivaptan effectively reduces depressive symptoms specifically in a subset of MDD patients, potentially identifiable by underlying HPA axis dysfunction. The confirmation of this hypothesis requires further studies integrating measures of HPA axis activity alongside response to nelivaptan treatment, facilitating precision psychiatry approaches for depression.

## 1 Introduction

Depression remains a major public health issue despite the availability of a large number of antidepressant medications and psychotherapeutic interventions, with major depressive disorder (MDD) the leading cause of disability worldwide (1).

The majority of currently used antidepressant medications modulate monoaminergic neurotransmission, with medications from the Selective Serotonin Reuptake Inhibitor (SSRI) and Serotonin-Norepinephrine Reuptake Inhibitor (SNRI) classes being the most common (2). Among the issues with these medications are relatively low response rates and low effect sizes (3, 4). Additionally, no assessment tools or clinical characteristics can reliably guide the clinician’s choice of treatment.

Disturbances in the function of the human stress response system, particularly the hypothalamus–pituitary–adrenal (HPA) axis, have been found in many depressed individuals (5–7). This triggered extensive efforts during the first decade of the millennium to develop modulators of the HPA axis function that could be putative antidepressants. A total of 12 inhibitors of the corticotropin-releasing hormone receptor 1 (CRHR1) and three inhibitors of the vasopressin V1b receptor were studied in Phase 2 MDD trials in humans (8). Despite this effort, no such medication ever reached regulatory approval. One possible explanation for this could be that the HPA axis dysfunction is thought to be present only in a subset of depressed individuals and that a positive effect in these individuals would be diluted in trials that also contained a large proportion of individuals with a normal HPA axis function (9).

Other vasopressin V1b receptor antagonists have been explored in major depression. For instance, ANC-501 (formerly TS-121), developed by Taisho and later by a U.S. biopharmaceutical company, demonstrated promising Phase 2 signals in patients with MDD (10). These efforts underscore a broader interest in targeting the HPA axis beyond nelivaptan.

**Figure 1:**
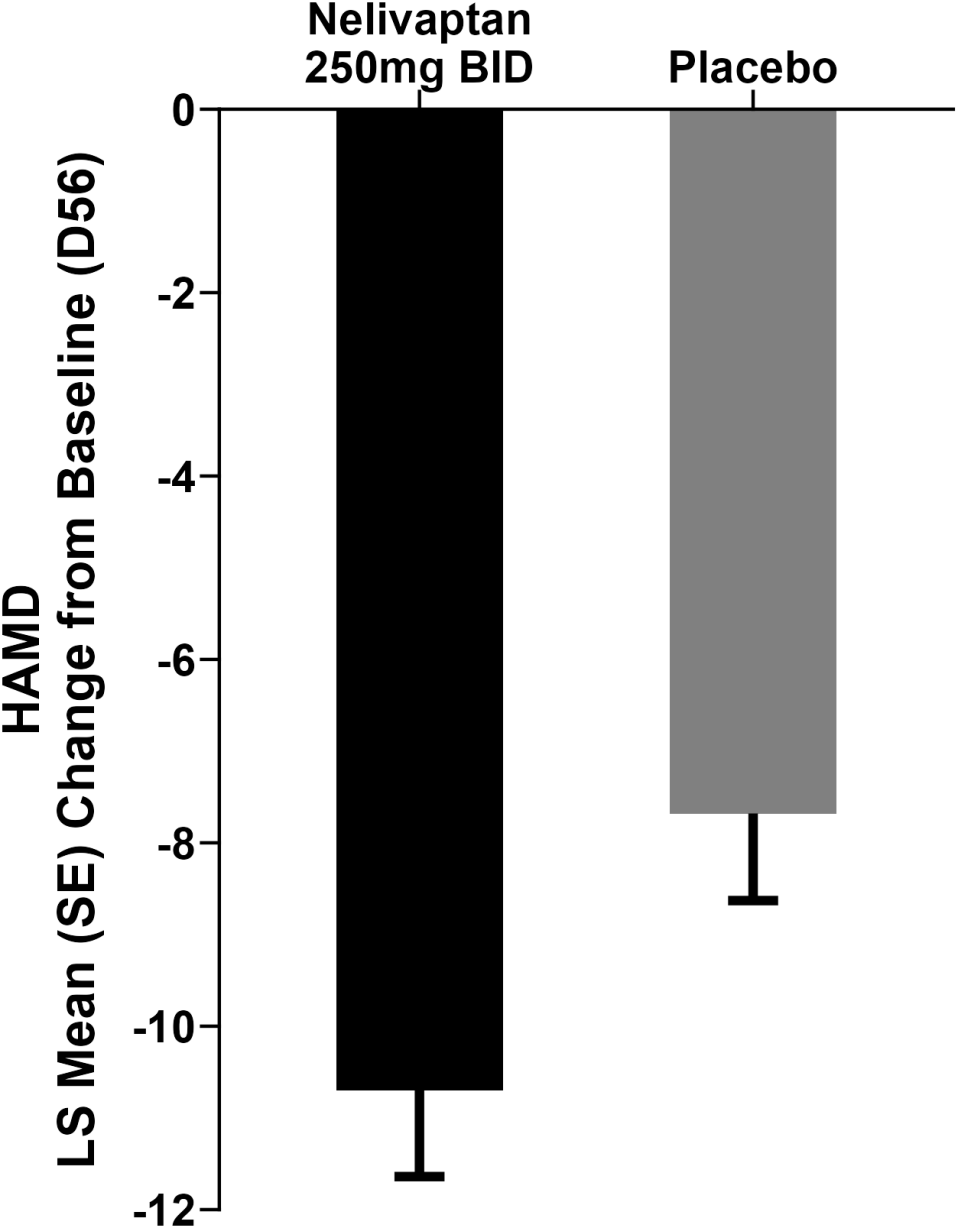
Results adapted from Griebel et al. (11). Change in HAMD from baseline to day 56: LS mean change from baseline in the active arm was −10.7 versus −7.68 in the placebo arm, p = 0.0244. BID, bis in die (i.e., twice daily dosing); HAMD, 17-item Hamilton Rating Scale for Depression; LS, least squares; MMRM, mixed model for repeated measures.

Nelivaptan is a potent and selective vasopressin V1b receptor antagonist that was studied in a total of 19 clinical trials in Phase 1 and Phase 2 as a potential treatment for MDD and generalized anxiety disorder (GAD) (11). In one of two Phase 2 MDD trials (NCT00358631), treatment with 250 mg BID nelivaptan resulted in a statistically significant separation in the 17-item Hamilton Rating Scale for Depression (HAMD) at the endpoint at day 56 (**Figure 1**) (11). The least squares (LS) mean change from baseline (SE) in the active arm was −10.7 (0.94) versus −7.68 (0.95) in the placebo arm, resulting in a difference from placebo (SE) of −3.03 (95% CI −5.66 to −0.39; p = 0.0244) in the original analysis (11). Cohen’s *d* effect size was 0.38. In the treatment arms that received 100 mg BID nelivaptan or 10 mg QD escitalopram, no statistically significant separation was detected. The program was, however, not pursued further.

There is renewed interest in HPA axis modulators as antidepressant treatments, this time in combination with predictive patient selection tools. A genetic companion diagnostics (CDx) is underway, integrating domain knowledge and physiological data from patients with MDD. The expectation is that approximately one-third of the depressed population will be identified using the CDx, indicating a high likelihood of an HPA axis dysfunction (12). A Phase 2 trial is underway, which explores the efficacy of nelivaptan and the performance of the CDx in MDD patients (13).

## 2 Methods

The data from trial NCT00358631 were reanalyzed with a focus on the HAMD measurements. Analyses were based on patients in the Intention-to-treat (ITT) set with a HAMD observation at day 56 [placebo arm (n = 63) and 250 mg BID nelivaptan arm (n = 62)]. Treatment response, expressed in changes in HAMD from baseline to day 56, for patients treated with 250 mg BID nelivaptan or placebo, was analyzed and modelled.

The main hypothesis in this investigation is whether the distribution of the treatment response, measured as HAMD change from baseline to day 56, y , in the active arm is a compound of two distributions, representing subsets of patients who do or do not respond to the treatment given. The distribution of response, f(y), in each arm separately, can be statistically defined as


(1) $$f(y)=\pi_{1}N(\mu_{1},\sigma_{1}^{2})+\pi_{2}N(\mu_{2},\sigma_{2}^{2}).$$

$N(\mu_{1},\sigma_{1}^{2})$ and $N(\mu_{2},\sigma_{2}^{2})$ represent two normal distributions with mean values $\mu_{1}$ and $\mu_{2}\;$ and variances $\sigma_{1}^{2}$ and $\sigma_{2}^{2}$ , respectively, and $\pi_{1}$ and $\pi_{2}$ represent the probability for a patient to be in latent classes 1 and 2, with $\pi_{1}+\pi_{2}=1$ .

**Figure 2:**
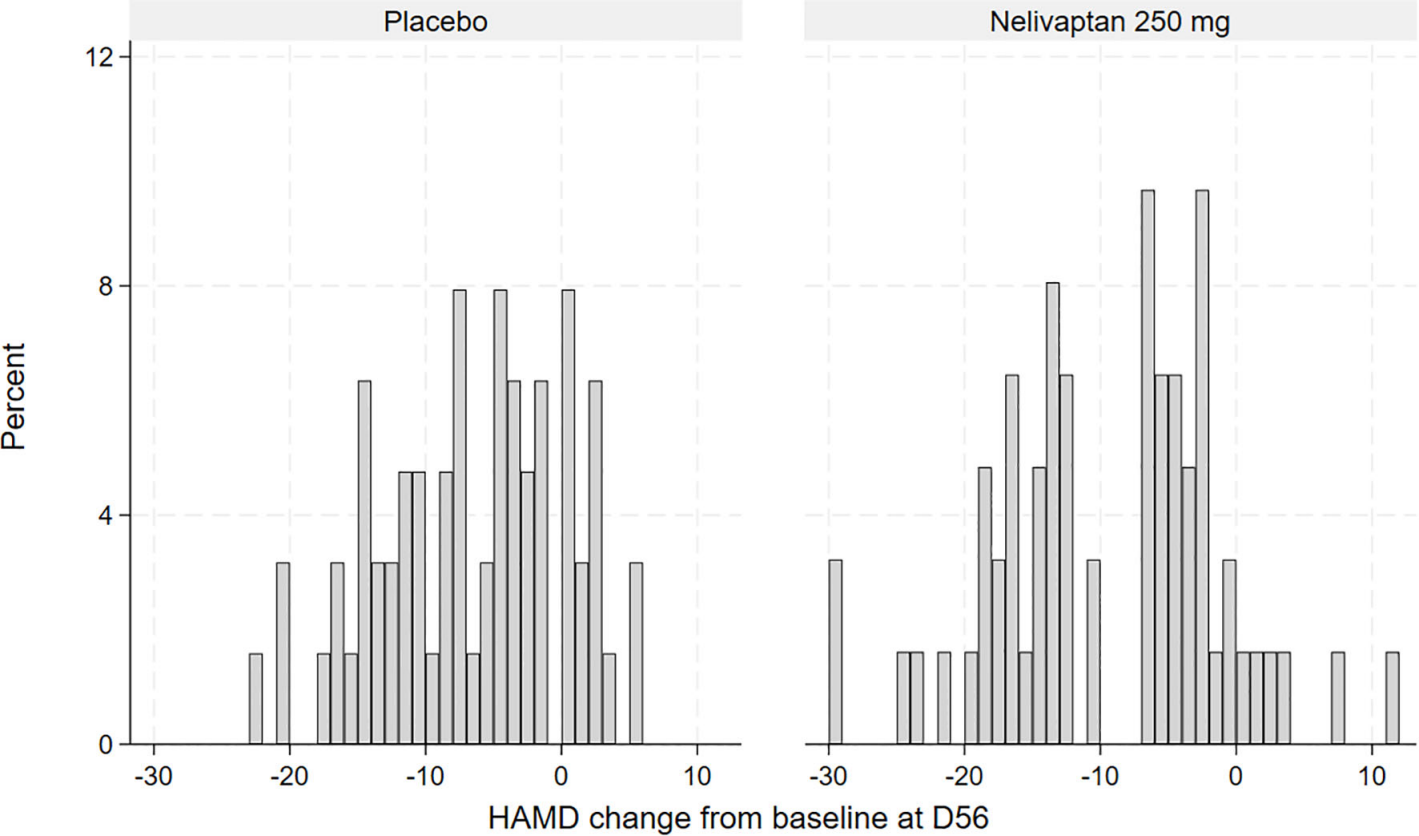
Histogram of observed HAMD change from baseline to day 56 (D56), placebo (n = 63), and 250 mg BID nelivaptan (n = 62, NCT00358631). HAMD, 17-item Hamilton Rating Scale for Depression.

To obtain a first impression of the distribution of treatment response, the HAMD changes from baseline to day 56 are displayed graphically using histograms in **Figure 2**. To analyze the main hypothesis statistically, we applied a finite mixture of linear regression models (FMMs) in the placebo arm and in the active arm, separately. The main concept of FMM is that the observed data come from two or more distinct, but unobserved (latent), subpopulations (14). In the present case, we hypothesized that there are two subpopulations with regard to response to nelivaptan, defined by their HPA axis dysfunction: the nelivaptan responders and non-responders. We also tested whether we could find subgroups within the placebo-treated patients, suggesting placebo responders and non-responders.

In general, we assume that the response in each of the latent classes follows a normal distribution, together forming a mixture of Gaussians. The baseline HAMD score was added as an adjusting covariate to the model, making $\mu_{1}$ and $\mu_{2}$ , in Equation 1, functions of the baseline HAMD score.

To also test whether the assumption of two subgroups fits the data of each treatment arm better than a model with three subgroups or just one group, we adapted different FMMs and compared the model fit using the Akaike information criterion (AIC) and Bayesian information criterion (BIC). After deciding on a certain number of subgroups, associations between the subgroups and demographic variables (sex or age) were checked within each arm.

## 3 Results

The frequencies of observed changes from baseline in the high-dose nelivaptan arm and the placebo arm are displayed in **Figure 2**. In the placebo arm, the distribution of change from baseline suggested normal distribution (**Figure 2**), while the distribution of improvement from baseline in the 250 mg BID nelivaptan arm appears to be bimodal or at least skewed. A similar but less pronounced distribution was seen for 100 mg BID nelivaptan (see **Supplementary Figure 1**).

Gaussian mixture models with different numbers of classes (one, two, or three classes) were calculated. The model fit criteria of the FMMs are displayed in **Table 1**. Both the AIC and BIC are criteria used to compare the model fit for the applied data in terms of the likelihood and the number of model parameters. The main difference is that the BIC penalizes model complexity in relation to the sample size more than the AIC.

**Table 1:** Goodness of fit from different finite mixture models.

Model	Nelivaptan 250 mg			Placebo		
	N	AIC	BIC	N	AIC	BIC
1 group	62	443	451	63	425	433
2 subgroups	62	437	452	63	425	440
3 subgroups	62	438	461	63	425	449

AIC, Akaike information criterion; BIC, Bayesian information criterion.

In the nelivaptan arm, the FMM with two subgroups is preferred by the AIC and, according to the BIC, only slightly worse than the model with only one group. In the placebo arm, all three models are similar in terms of the AIC, but the one-group model fits best according to the BIC.

The FMM for the nelivaptan arm suggested two normal distributions with means (SE) −17.14 (0.96) and −3.85 (1.58). The two classes contained 55% and 45% of the mass, calculated as the marginal probabilities of the patients being in each arm.

In the high-responder class, the HAMD measurement at baseline has a significant association with a parameter estimate of −1.16 (95% CI −1.66 to −0.65; p < 0.001), whereas there is no association in the low-response subgroup. The likelihood of each patient being in each class is not associated with age or sex.

**Figure 3:**
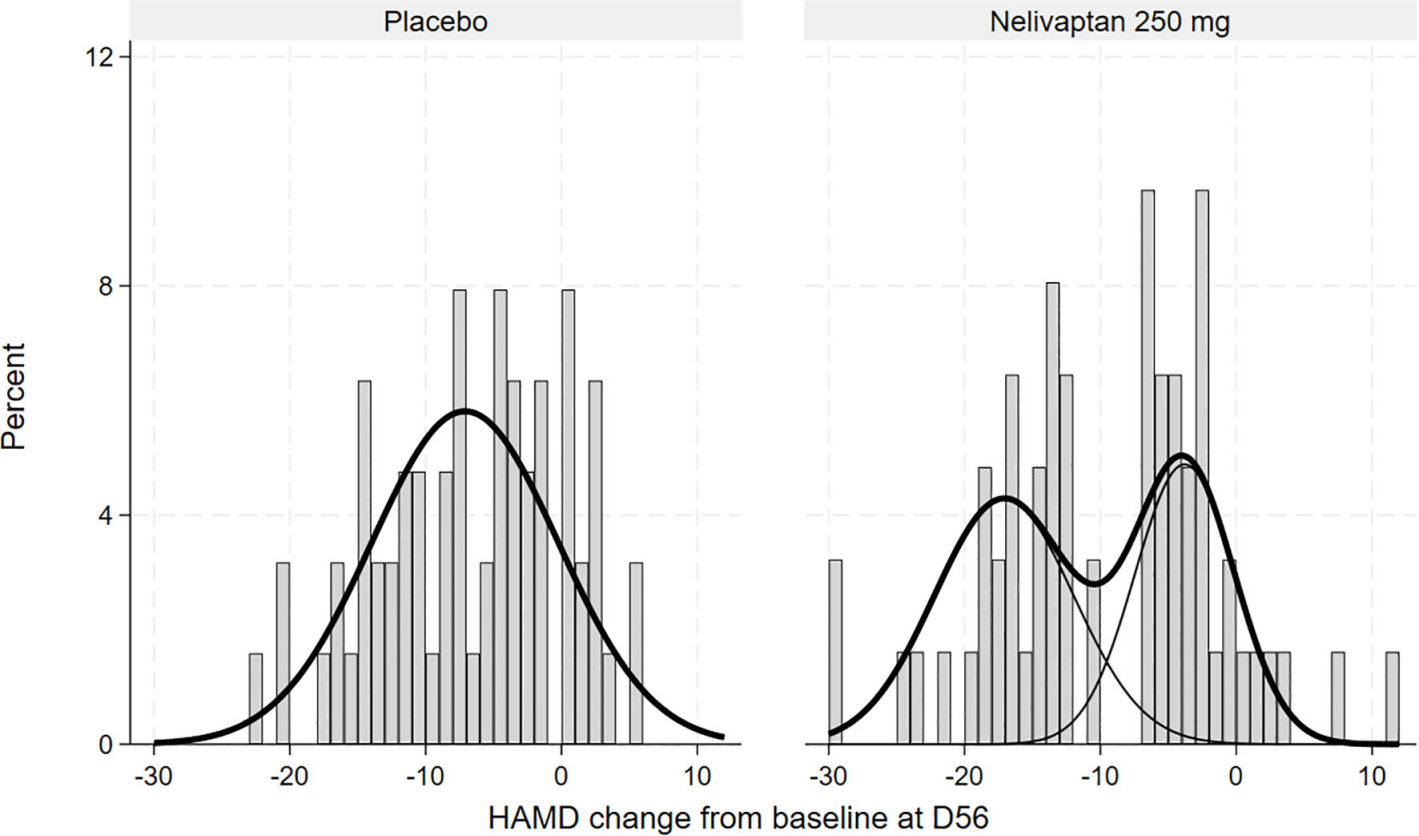
Histogram of HAMD changes from baseline to day 56 in the placebo (n = 63) and nelivaptan (n = 62) arms at day 56, with estimated unimodal and bimodal distributions (NCT00358631). HAMD, 17-item Hamilton Rating Scale for Depression.

The FMM for the placebo arm suggested just one normal distribution with means (SE) −7.06 (0.86), as displayed in **Figure 3**.

## 4 Discussion

Our reanalysis provides statistical evidence suggesting a bimodal distribution of antidepressant response among patients treated with 250 mg BID nelivaptan, distinguishing two subpopulations: high responders and low responders. Notably, the identified high-responder subgroup, comprising approximately 55% of patients, aligns well with the previously reported overall response rate of 45.7% for nelivaptan at this dosage. This congruence strengthens the plausibility of a biologically distinct subgroup responsive to nelivaptan treatment.

The observed bimodality supports the hypothesis that vasopressinergic modulation, specifically via V1b receptor antagonism, seems effective predominantly in patients who have underlying disturbances of the HPA axis. This interpretation is consistent with extensive prior research linking depressive symptoms to dysregulated HPA axis activity (5–7). The absence of such a bimodal distribution in the placebo group suggests the specificity of nelivaptan’s mechanism of action, potentially explaining why previous attempts to establish the efficacy of HPA axis modulators in broader patient populations were unsuccessful (8, 9).

A limitation of the current analysis is the lack of analysis of direct biological measures of HPA axis activity, such as cortisol levels or other neuroendocrine biomarkers, which prevents the definitive identification of the biological mechanisms underlying the observed response patterns. Moreover, this study analyzed data from only one clinical trial, and the pronounced bimodality observed in the 250 mg BID nelivaptan arm was not equally apparent at the lower dose of 100 mg BID (**Supplementary Figure 1**), suggesting dose dependence of this response pattern. While our study highlights the importance of biological stratification, several *in vivo* measures of HPA axis activity have been proposed, including the dexamethasone suppression test, late-night salivary cortisol, 24-h urinary free cortisol, plasma adrenocorticotrophic hormone (ACTH), and plasma copeptin (a surrogate for arginine vasopressin) (15, 16). However, these tests can be variable and affected by stress, circadian rhythms, and medications. The CDx targeting V1b receptor signaling may offer a more stable, trait-level marker of HPA axis dysregulation. An integrated approach—combining genetic predictors with dynamic endocrine testing—could provide a robust precision psychiatry framework, increasing the likelihood of identifying true responders to V1b receptor antagonism.

To validate and extend these findings, future studies should incorporate a biological assessment of HPA axis activity to confirm the hypothesized mechanism. The ongoing OLIVE trial (EudraCT number: 2022-002757-26) addresses this directly, testing nelivaptan efficacy in conjunction with a genetic companion diagnostic tool designed to predict vasopressin V1b receptor signaling disturbances. Positive outcomes from such studies would represent significant progress toward implementing precision psychiatry approaches, enabling tailored therapeutic strategies based on biological subtype identification in major depressive disorder.

## Data Availability

The datasets presented in this article are not readily available because they cannot be shared due to contractual restrictions between HMNC Holding GmbH and Sanofi. Requests to access the datasets should be directed to christine.eulenburg@hmnc-brainhealth.com.

## Supplementary material

Supplementary Figure 1 Histogram of observed HAMD change from baseline at day 56 (D56), nelivaptan 100 mg BID (n = 64, NCT00358631).
