# Supplementary figures and images for: Toward precision psychiatry: theoretical implications of bimodal response patterns to vasopressin V1b receptor inhibition in depression
Source: Front Psychiatry. 2025 Oct 16;16:1645225. doi: 10.3389/fpsyt.2025.1645225 (PMC12573134; doi:10.3389/fpsyt.2025.1645225)

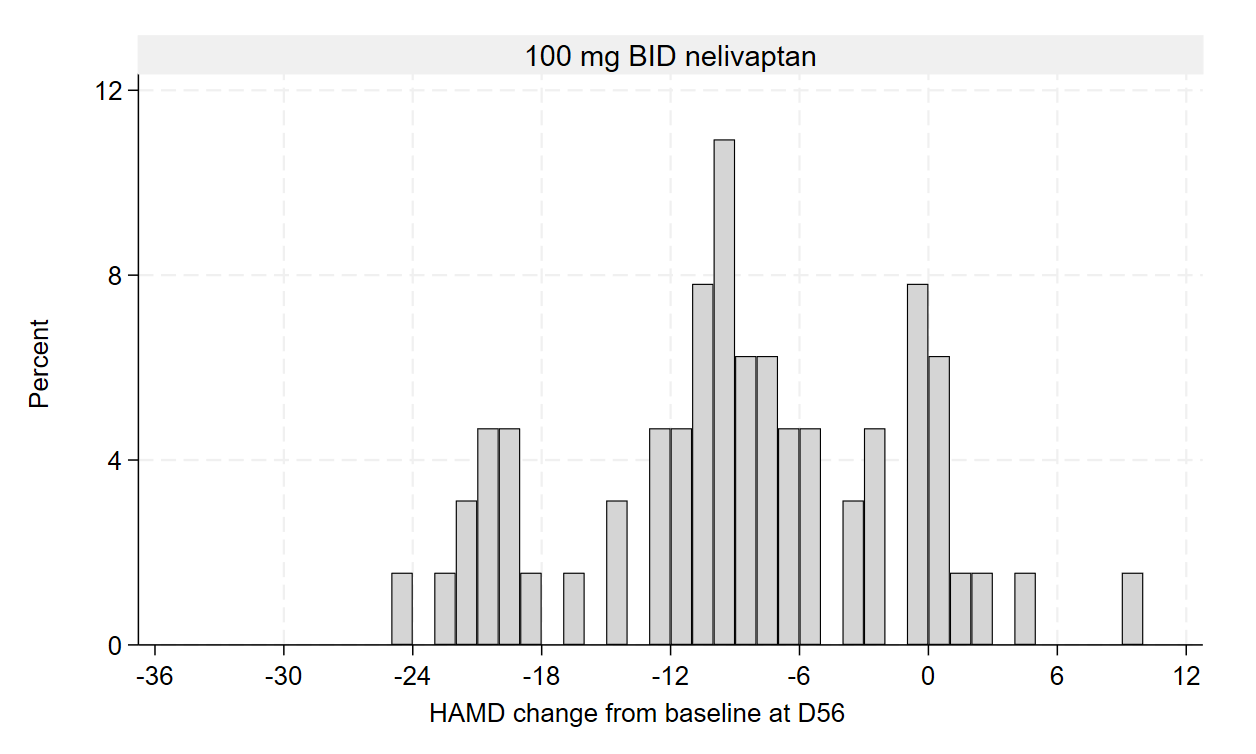

Supplement: Supplementary Figure 1 — Histogram of observed HAMD change from baseline at day 56 (D56), nelivaptan 100 mg BID (n = 64, NCT00358631). [file Image1.tif]
